# Optimizing Preoperative Anemia in Non-Metastatic Colorectal Cancer: A Systematic Review on Surgical Recovery and Outcomes

**DOI:** 10.3390/cancers17223689

**Published:** 2025-11-18

**Authors:** Sophia Tsokkou, Ioannis Konstantinidis, Menelaos Papakonstantinou, Paraskevi Chatzikomnitsa, Areti Danai Gkaitatzi, Eftychia Liampou, Antonios Fantakis, Georgia Kolympa, Evdokia Toutziari, Dimitrios Alexandrou, Dimitrios Giakoustidis, Petros Bangeas, Vasileios N. Papadopoulos, Alexandros Giakoustidis

**Affiliations:** 1 First Department of Surgery, General Hospital Papageorgiou, Aristotle University of Thessaloniki, 56429 Thessaloniki, Greece; menelaospap.md@gmail.com (M.P.); voula.hatzikomnitsa@yahoo.gr (P.C.); aretidanaegtz24@gmail.com (A.D.G.); liampouef7@yahoo.gr (E.L.); antfandakis@yahoo.gr (A.F.); georgettekolympa@gmail.com (G.K.); evdo-t@hotmail.com (E.T.); alexandrousurgeon@gmail.com (D.A.); dgiak@auth.gr (D.G.); pmpangeas@auth.gr (P.B.); papadvas@auth.gr (V.N.P.); 2 Department of Medicine, Faculty of Health Sciences, Aristotle University of Thessaloniki, 54124 Thessaloniki, Greece; ikonsc@auth.gr

**Keywords:** preoperative anemia, colorectal cancer, non-metastatic colorectal cancer, surgical outcomes, surgical recovery, perioperative management, hemoglobin optimization, iron deficiency anemia, blood transfusion, erythropoiesis-stimulating agents, systematic review, risk stratification, treatment efficacy

## Abstract

Background/Objectives: Colorectal cancer (CRC) is among the most commonly reported malignancies globally, taking the third place in incidence among males as well as the second among females, with over 1.9 million new cases and 935,000 deaths estimated worldwide in 2020. One of the most common and clinically significant comorbidities in patients undergoing CRC surgery is preoperative anemia, with reported prevalence ranging from 23% to over 60% depending on the population and diagnostic criteria used. The objective of the current study is to systematically evaluate the current body of evidence on preoperative anemia management in adults undergoing surgery for non-metastatic CRC. This review aims to assess the clinical impact of different iron supplementation strategies, particularly IV versus oral iron in pre-operative hematologic optimization, transfusion requirements, postoperative complications, and recovery outcomes. Methods: The current systematic review was conducted based on the PRISMA for Systematic Reviews and Meta-analysis checklist. The study has been registered to PROSPERO with the ID CRD420251113455. Results: Across all studies, IV iron and erythropoiesis-stimulating agents emerged as safe and more efficient alternatives to iron per os, especially when initiated at least two weeks before surgery. Thus, highlighting the clinical value of proactive anemia management as a cornerstone of surgical prehabilitation, potentially reducing transfusion burden and improving recovery outcomes for CRC patients. Conclusions: It is observed that IV iron therapy offers a more effective strategy than per os supplementation for correcting preoperative anemia in non-metastatic colorectal cancer patients. Their hematologic benefits enhance surgical readiness and reduce postoperative intervention needs. Thus, supporting the integration of IV iron into preoperative optimization protocols.

## Simple Summary

Colorectal cancer is among the most commonly reported malignancies globally, taking the third place in incidence among males as well as the second among females, with over 1.9 million new cases and 935,000 deaths estimated worldwide in 2020. The current systematic review was conducted based on the PRISMA for Systematic Reviews and Meta-analysis checklist. The study has been registered to PROSPERO with ID CRD420251113455. Across all studies, IV iron and erythropoiesis-stimulating agents emerged as safe and more efficient alternatives to iron per os, especially when initiated at least two weeks before surgery. Their hematologic benefits enhance surgical readiness and reduce postoperative intervention needs. Thus, supporting the integration of IV iron into preoperative optimization protocols.

## 1. Introduction

Colorectal cancer (CRC) is among the most commonly reported malignancies globally, taking the third place in incidence among males as well as the second among females, with over 1.9 million new cases and 935,000 deaths estimated worldwide in 2020 [1]. Surgical resection remains the primary curative approach for CRC, and is often accompanied by neoadjuvant or adjuvant therapies, which depend on tumor stage and location (left colon, right colon, or rectum) [2,3]. It is crucial to understand the risks of the perioperative period, as it encompasses the time around a surgical procedure, carries significant risks that can affect both short-term and long-term patient outcomes [4,5]. Such risks include complications related to anesthesia, surgical procedures, and pre-existing medical comorbidities, as well as potential psychological impacts like anxiety [4,6].

One of the most common and clinically significant comorbidities in patients undergoing CRC surgery is preoperative anemia, with reported prevalence ranging from 23% to over 60% depending on the population and diagnostic criteria used [7]. Iron deficiency anemia is the most reported form of anemia in CRC patients, and up to 88% of all anemic CRC patients have been diagnosed with iron deficiency anemia [8]. Anemia in CRC is of multifactorial cause, arising from a combination of factors, of which chronic gastrointestinal blood loss is a primary cause, leading to iron deficiency [9]. In addition, nutritional deficiencies, especially in iron, folate, and vitamin B12, are common [10,11]. Systemic inflammation associated with the cancer and its treatment is also a contributor, as well as bone marrow suppression caused by the malignancy itself or prior therapies undertaken [11,12]. More specifically, inflammation associated with malignancy can lead to functional iron deficiency via the hepcidin pathway, whereas chronic blood loss causes absolute iron deficiency and depletion of iron stores [13].

**Figure 1:**
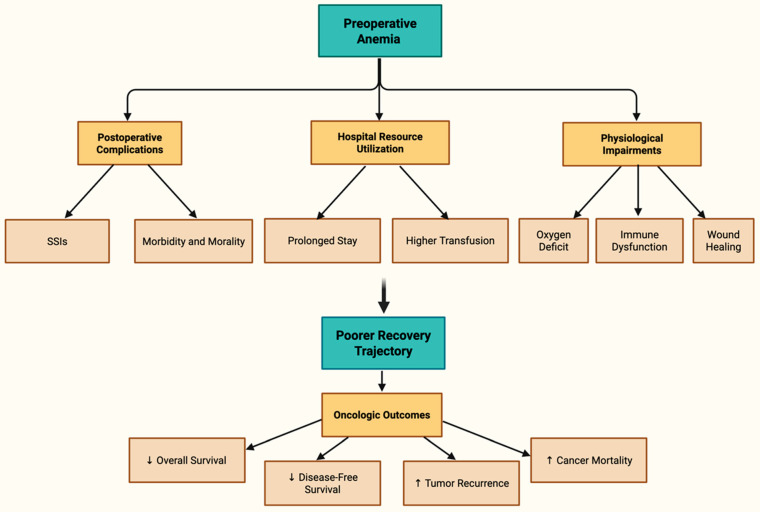
Tree Diagram of Preoperative Anemia Impact [14,15,16].

Figure 1 comprehensively illustrates the multidimensional impact of preoperative anemia, branching into key clinical domains including postoperative complications, hospital resource utilization, physiological impairments, overall surgical recovery, and oncologic outcomes [14,15,16].

In accordance with the World Health Organization (WHO), anemia is diagnosed based on hemoglobin concentrations that vary by sex, age, and physiological status [17]. The classification criteria for anemia are comprehensively outlined in Table 1.

Serum ferritin (SF) serves as a key biomarker for assessing iron status, with levels below 15 µg/L indicating iron deficiency in adults. However, as an acute-phase reactant, SF thresholds must account for inflammation [18]. For instance, in the presence of elevated CRP (>5 mg/L), a higher cutoff of <200 µg/L combined with transferrin saturation (TSAT) <13% improves diagnostic accuracy (Table 2) to prevent the underdiagnosis of iron insufficiency in patients with simultaneous inflammatory conditions [19], like CRC.

TSAT represents the percentage of transferrin bound to iron and is calculated using serum iron and total iron-binding capacity (TIBC). A TSAT below 20% is an indication of iron deficiency, while values above 50% suggest iron overload. TSAT is useful for distinguishing iron deficiency anemia from anemia of chronic disease when interpreted with ferritin levels (Table 3) [20].

Notwithstanding these findings, the present literature exhibits heterogeneity in definitions of anemia, study methods, outcome measures, and patient demographics. Certain studies indicate that preoperative correction of anemia, especially with intravenous (IV) iron, may reduce these risks, such as Wilson et al. (2018) who found a marked hemoglobin elevation linked to potentially fewer complications [21], although others claim minimal advantage, as evidenced by the Cochrane review by Ng et al. (2019) which reports uncertain reductions in transfusions among low-quality evidence [22]. Barsballe et al. (2024) further illustrate this tension, showing hemoglobin increase but incomplete anemia resolution in many cases [23], while Fritche et al. (2025) suggest benefits in length of stay, yet highlight persistent transfusion need in untreated groups [24]. Muñoz et al. (2014) emphasize IV iron’s role but note variability tied to underlying iron deficiency types [25]. The relevance of perioperative transfusions as a confounding or mediating factor remains contentious, with some studies indicating immunosuppressive effects that could exacerbate oncologic outcomes. Evidence from both trauma and elective gastrointestinal surgery demonstrates that allogeneic transfusion is associated with a gene expression profile characteristic of immunosuppression, including reduced Th1/Th17 activity and increased IL-10, even when leukodepleted products are used, and this correlates with higher rates of postoperative infection [26,27]. At the same time, anemia itself contributes to impaired immune competence and tissue oxygenation, suggesting that both anemia and transfusion act as interdependent drivers of adverse surgical and oncologic outcomes. Due to the clinical significance and potential for modification of preoperative anemia, a thorough synthesis of the current research is necessary. Thus, this systematic analysis aims to thoroughly examine the relationship between preoperative anemia and postoperative outcomes in adults following CRC surgery.

## 2. Materials and Methods

The current systematic review was conducted based on the PRISMA for Systematic Reviews and Meta-analysis checklist (Table S1). The study has been registered to PROSPERO on the 27 July 2025 with ID CRD420251113455.

### 2.1. Objectives

The objective of the current study is to systematically evaluate the current body of evidence on preoperative anemia management in adults undergoing surgery for non-metastatic CRC. The present review aims to assess the clinical impact of different iron supplementation strategies, particularly IV versus oral iron in pre-operative hematologic optimization, transfusion requirements, postoperative complications, and recovery outcomes. By consolidating data from randomized controlled trials (RCTs), the review seeks to inform best practices in surgical prehabilitation and support evidence-based enhancements to perioperative care pathways for CRC patients.

### 2.2. Eligibility Criteria

For the eligibility criteria, the Population, Intervention, Comparator, Outcome (PICO) framework was used as shown in Table 4. The inclusion and exclusion criteria as mentioned in Section 2.2.1 and Section 2.2.2.

#### 2.2.1. Inclusion Criteria

- M0-Staged Colorectal Carcinoma Patients;
- Pre-operative administration of Iron Supplementation (Oral or IV);
- Peer-reviewed primary studies and reviews addressing the research question;
- Studies published in English;
- Randomized Controlled Trials (RCTs);
- Adults (≥18 years).

#### 2.2.2. Exclusion Criteria

- Metastatic CRC patients;
- Post-operative administration of Iron Supplementation without prior pre-operative administration;
- Editorials, commentaries, opinion pieces, cohort studies, case–control, observational.
- Animal studies;
- Articles without full-text access;
- Non-English Papers;
- Pre-prints.

### 2.3. Search Strategy

An exhaustive search was conducted across PubMed (MEDLINE), Scopus, ScienceDirect, and Cochrane Library using a combination of MeSH terms and keywords such as “colorectal cancer”, “preoperative anemia”, “postoperative outcomes”, and “surgical complications”. Search results were exported for screening, and duplicates were removed.

### 2.4. Study Selection and Data Charting

Two reviewers (S.T. and I.K.) independently screened titles and abstracts based on the eligibility criteria, followed by full-text screening. Discrepancies were resolved through a third reviewer (P.C.). A standardized data extraction form was developed to chart key information using Microsoft 365 Excel software, including (a) Study characteristics: author, year, country, design, (b) Population details: sample size, age, gender, cancer type/stage, (c) Anemia definition and measurement, (d) Postoperative outcomes assessed, and (e) Key findings and limitations. Although a meta-analysis was considered, pooling of results was deemed inappropriate due to substantial clinical and methodological heterogeneity across studies, including differences in study design, populations, interventions, outcome definitions, and reporting metrics. Thus, the findings were synthesized narratively and organized thematically to highlight areas of consensus and evidence gaps.

### 2.5. Quality Assessment

For the methodological quality assessment and potential risk of bias evaluation across the included studies, two complementary evaluation tools were utilized, including the Jadad Scale and the Cochrane Risk of Bias 2 (RoB 2) framework. Each domain was independently reviewed, with judgments categorized as low risk, some concerns, or high risk, facilitating a nuanced understanding of each study’s limitations. By combining these tools, a robust and multidimensional appraisal of trial quality was ensured, which informed subsequent inclusion decisions and interpretations within the synthesis framework.

## 3. Results

### 3.1. Study Selection

**Figure 2:**
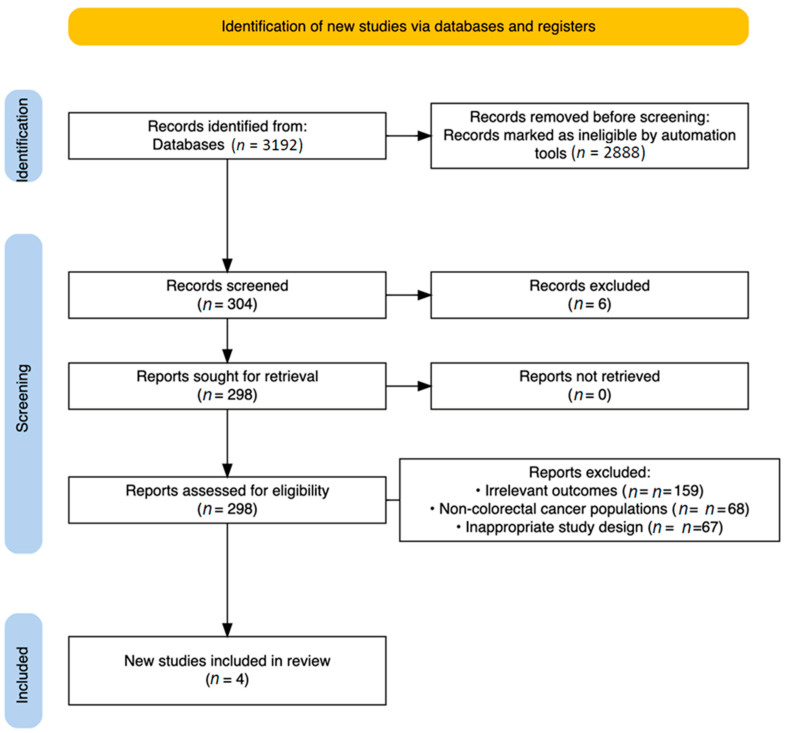
PRISMA Flow Diagram.

A comprehensive search of four major databases yielded a total of 3192 articles, with the following distribution. From PubMed (*n* = 70), Scopus (*n* = 76), ScienceDirect (*n* = 3042), and the Cochrane Library (*n* = 4), these articles were identified (Figure 2).

Following the automation-assisted filtering, 2888 records were eliminated, leaving 304 for full text screening, after the de-duplication process (*n* = 6). From those, exclusions were made for the following reasons:

- Irrelevant outcomes (*n* = 159);
- Non-colorectal cancer populations (*n* = 68);
- Inappropriate study design, primarily non-randomized studies (*n* = 67).

Ultimately, four studies met all eligibility criteria and were included in the final analysis [21,22,23,24].

### 3.2. Quality Assessment Outcomes

Methodological quality was evaluated using both the Jadad Scale (Table 5) and the Cochrane Risk of Bias 2 (RoB 2) (Table 6) framework. Even though methodological variation existed among trials, each study presented sufficient transparency and validity in outcome reporting. The incorporation of objective endpoints and clearly defined inclusion criteria across studies enhances the reliability of this comparative synthesis.

### 3.3. Main Findings

The four RCTs collectively examined strategies for managing iron deficiency anemia prior to colorectal cancer surgery. Across all studies, IV iron and erythropoiesis-stimulating agents emerged as safe and more efficient alternatives to oral iron, especially when initiated at least two weeks prior to resection. The findings stress the positive clinical value of proactive anemia management as a cornerstone of surgical prehabilitation, potentially reducing transfusion burden and improving recovery outcomes for CRC patients. The findings from each included study are comprehensively charted in Table 7, providing a consolidated overview of the interventions and their impact on anemia management in the context of surgical prehabilitation.

### 3.4. Specific Resection Sites and Surgical Approach

Table 8 illustrates that the laparoscopic technique consistently prevailed across trials, especially among patients undergoing IV iron therapy, a trend most pronounced in the Talboom and Fung studies [28,30], when laparoscopic surgery achieved rates as high as 91%. Right hemicolectomy was the predominant resection, particularly in the FIT and IVICA trials, with oral iron groups often exhibiting a slightly elevated count. IV iron recipients exhibited a reduced incidence of “no resection” surgeries, indicating enhanced tumor resectability, possibly associated with improved physiological condition. Simultaneously, Christodoulakis’s EPO study [31] concentrated on tumor localization rather than procedural classification, emphasizing an equitable distribution across left-sided, right-sided, and rectal malignancies. The data indicate that IV iron can enhance surgical outcomes by facilitating more comprehensive resections, hence highlighting its significance in perioperative management for iron-deficient CRC patients.

## 4. Discussion

By synthesizing data from four RCTs, the systematic review compared IV versus oral iron supplementation, as well as erythropoiesis-stimulating agents, to identify interventions most likely to improve perioperative status and recovery.

Firstly, Christodoulakis et al. (2005) evaluated the effect of preoperative epoetin alfa on transfusion requirements in anemic patients undergoing CRC resection, the administration of high-dose epoetin alfa (300 IU/kg/day) for 10 days prior to the resection had led to significant preoperative elevation of hemoglobin with a *p*-value of 0.008 and hematocrit *p*-value of 0.0005, with sustained postoperative enhancements of *p* = 0.011 and *p* = 0.0008, respectively [31]. The regimen was correlated with a notable decrease in perioperative transfusion requirements, with a mean of 0.81 vs. 1.34 units and *p* = 0.016, and postoperative transfusion needs with a mean of 0.87 vs. 1.35 units and *p* = 0.023 relative to controls. Conversely, a reduced dosage of 150 IU/kg/day produced slight hematological enhancements without a statistically significant decrease in transfusions, with *p* > 0.05. Both epoetin groups demonstrated significantly reduced ferritin levels at baseline with *p* < 0.05, indicative of iron use, with recovery by day +15. While transferrin saturation (TSAT) values were not explicitly provided, the patterns in serum iron and transferrin indicated vigorous erythropoiesis. No significant adverse events were associated with epoetin alfa, supporting its safety and tolerability. The data suggest that high-dose epoetin alfa may be a useful preoperative method to enhance hematological status and reduce transfusion reliance, especially in circumstances with restricted time for prehabilitation [31].

Alongside the previous results, the IVICA multicenter RCT examined IV ferric carboxymaltose versus oral ferrous sulfate in anemic patients with non-metastatic CRC scheduled for elective surgery [29]. Although there was no significant difference in transfusion volume during surgery between groups (*p* = 0.841), IV iron produced a greater median increase in hemoglobin preoperatively (1.55 g/dL vs. 0.50 g/dL; *p* < 0.001). Additionally, fewer patients were anemic at the time of surgery (75% vs. 90%; *p* = 0.048), underscoring better optimization. Moreover, IV iron produced notably greater increases in ferritin and TSAT than oral iron (*p* < 0.001 for both), suggesting a better replenishment of systemic iron stores. Also, the need for postoperative iron supplementation was considerably lower in the IV group (4 vs. 30 patients; *p* < 0.001). Adverse effects were negligible in both the IV iron and oral iron groups, emphasizing the safety of the IV treatment. Although the transfusion rate did not change, the biochemical and clinical benefits support the use of IV iron as an alternative, especially when a rapid correction is needed, or in cases of poor absorption of oral iron. These results also support the need to assess treatment benefits in context, but not limited to, practical considerations regarding cost, access to treatment, and timeliness. The study revealed a lower prevalence of “no resection” cases in the IV cohort, supporting a potential physiological build that could allow a higher percentage of total resection of the tumor. The link between treatment of anemia and surgical viability deserves further exploration in future studies [29].

The FIT trial reinforces the postoperative utility of IV iron. Specifically, the trial found that 30 days post-surgery, hemoglobin normalization had occurred in 60% of patients receiving IV iron in comparison to 21% for the oral cohort (*p* < 0.0001), thus demonstrating a statistically significant and clinically meaningful difference [30]. On the day of surgery, hemoglobin normalization was similarly low in both patient cohorts (17% vs. 16%; *p* = 0.83), which suggests that neither iron treatment approach was able to achieve sufficient correction in the limited period leading up to surgery. This finding reinforces the importance of time and might even foster the notion of delaying surgery in certain cases to maximize the hematologic potential of IV iron. In general, the biochemical results were somewhat more favorable for the IV iron group with greater increases in TSAT (approximately 25% vs. 10%) and ferritin (around 80 ng/mL vs. 30 ng/mL) levels. Subgroup analysis indicated the potential for enhanced perioperative outcomes for patients with mild anemia, including reduced ICU admission rates (5% vs. 12%) and lower rates of reintervention (3% vs. 10%), although these were not statistically significant (*p* ≈ 0.06–0.08) [30]. In summary, the FIT findings provide further support for consideration of IV iron into multimodal prehabilitation programs, where the surgical schedule permits opportunity for preoperative optimization.

Adding to the body of evidence, the pilot RCT examined preoperative administration of IV iron isomaltoside in patients with CRC and iron deficiency anemia (IDA), demonstrating significant improvements in hemoglobin (+6.1 g/L; *p* = 0.040) and ferritin (+297 µg/L; *p* < 0.001) compared with controls [28]. These effects were achieved within a median preoperative interval of 23 days, indicating the rapid biochemical efficacy of isomaltoside. While transfusion rates were lower in the intervention arm (10% vs. 30%), the difference did not reach significance, likely due to the limited sample size (*n* = 40). No differences were observed in postoperative complications, length of stay, or quality of recovery (QoR--15) scores, and the treatment was well tolerated without reported infusion-related adverse events. Nevertheless, slow recruitment rates in this study point to practical challenges in scaling up similar protocols. As a result, while the hematological benefits are evident, further adequately powered multicenter trials are required to clarify clinical outcome improvements and confirm the role of iron isomaltoside in routine preoperative practice [28].

Analysis of the results from the four RCTs indicates that IV iron consistently surpassed oral supplementation in enhancing hematological indices, hemoglobin, ferritin, and transferrin saturation within constrained preoperative periods [28,29,30,31]. High-dose epoetin alfa was distinctly linked to substantial decreases in transfusion needs, indicating a potential role for combination or sequential treatment approaches. Trends in the trials suggest possible correlations between enhanced hematologic status and surgical viability, encompassing increased resectability and wider adoption of laparoscopic techniques [28,29,30,31].

Our findings align with several prior systematic reviews and meta-analyses on preoperative anemia management in CRC patients, yet they also reveal persistent gaps in the evidence base that warrant a more nuanced interpretation. For instance, Sydhom et al.’s 2024 [32] meta-analysis of 11 studies demonstrated that preoperative intravenous (IV) iron significantly elevated hemoglobin levels at key perioperative time points and reduced transfusion rates, particularly postoperatively, mirroring our observation of IV iron’s superiority over oral supplementation in hematologic optimization and decreased transfusion needs. Similarly, Tang et al.’s 2022 [33] meta-analysis of seven trials reported a pooled increase in hemoglobin (mean difference 0.41 g/dL) and a 40% reduction in transfusion risk with iron supplementation, reinforcing the efficacy we identified in non-metastatic CRC cases. However, these studies, like Borstlap et al.’s 2015 [34] review of seven heterogeneous trials, often failed to consistently assess postoperative morbidity or long-term outcomes, leading to inconclusive evidence on broader surgical recovery, areas where our review advances by explicitly evaluating these endpoints. Extending this synthesis, our emphasis on non-metastatic CRC patients highlights prognostic implications that prior analyses have underexplored. Wilson et al.’s 2017 [35] meta-analysis of 12 studies linked preoperative anemia to poorer overall survival (hazard ratio 1.56) and disease-free survival (1.34), particularly in rectal cancer, which echoes Chardalias et al.’s 2023 [13] narrative review cautioning against anemia’s role in exacerbating inflammation via hepcidin pathways and increasing transfusion-related risks. Yet, these reviews predominantly drew from retrospective data with high heterogeneity, potentially inflating associations without accounting for confounding factors like tumor stage or neoadjuvant therapies, limitations which our focused review mitigates by restricting to non-metastatic disease and prioritizing RCTs, thereby offering a clearer lens on how anemia correction might influence recovery without advanced malignancy and metastases as a potential cofounding factor.

Certain limitations exist despite the strict methodology employed. Preoperative anemia in CRC is a highly prevalent and clinically significant problem, yet the randomized evidence-based addressing its management remains surprisingly sparse. The fact that only four RCTs met our inclusion criteria underscores this gap, limiting generalizability and statistical power. Rather than diminishing the relevance of the review, however, this scarcity highlights the urgent need for further well-designed multicenter trials.

The included RCTs exhibit substantial heterogeneity in interventions and comparators, with one trial evaluating epoetin alfa plus iron against iron alone, two comparing IV versus oral iron, and another assessing IV iron against no treatment. This diversity precluded meta-analysis and necessitated a narrative synthesis. While this approach sacrifices quantitative precision, it avoids the false authority of a pooled estimate that would almost certainly have been misleading. Instead, the synthesis highlights recurring themes such as hematologic improvement and reduced transfusion requirements without collapsing disparate interventions into a single effect size. Thus, the absence of a meta-analysis reflects the heterogeneity of the available evidence rather than a methodological weakness of the review.

Additional limitations include variability in treatment initiation times, dosing regimens, and outcome definitions, which introduce confounding and reduce comparability across studies. Most trials were conducted in specialized centers, which may constrain generalizability to broader healthcare settings with different resource availability. Although risk of bias was formally assessed, potential publication bias cannot be excluded, particularly given the predominance of trials reporting favorable hematologic outcomes. Finally, the reliance on short-term perioperative outcomes restricts conclusions regarding long-term oncologic endpoints such as recurrence or survival.

Taken together, these limitations emphasize the need for greater standardization in future research and underscore the importance of larger, multicenter RCTs with harmonized definitions and longer follow-up to confirm the role of intravenous iron and erythropoiesis-stimulating agents in preoperative optimization for colorectal cancer surgery.

## 5. Conclusions

Preoperative anemia remains a highly prevalent, clinically significant, and modifiable comorbidity among patients undergoing surgery for non-metastatic colorectal cancer. IV iron therapy, alone or with erythropoiesis-stimulating agents, consistently improves hematological responses compared to oral iron supplementation, according to the studies examined. Increases in hemoglobin concentration, ferritin, and transferrin saturation improve preoperative physiological optimization, reduce the need for postoperative iron supplementation, and sometimes increase surgical feasibility and access to minimally invasive techniques. When paired with iron, high-dose epoetin alfa reduced transfusion dependency and increased hematocrit, suggesting a role for multimodal correction. The therapies were safe and tolerable, with few side effects, validating their use in enhanced recovery pathways. The limited levels of hemoglobin normalization before surgery in several trials emphasize the necessity of early anemia correction and, when oncologically feasible, adjusted surgical schedules to optimize results. Due to the methodological diversity and small sample size of the studies included in this review, further multicenter randomized controlled trials will be needed to assist with generalizability, confirm long-term oncologic outcomes, and determine the most effective dosing and timing. Cost-effectiveness analysis will also be required to determine the economic impact of using IV iron and erythropoiesis-stimulating agents as part of the preoperative pathway, especially in resource-constrained settings for oncologic patients. These studies would also be useful in developing evidence-based guidelines for practitioners, which would allow for more widespread adoption of these interventions in clinical settings.

## Figures and Tables

**Table 1:** Definition of anemia based on hemoglobin concentrations (in g/dL), according to the WHO [17].

Population Group	Non-Anemic	Mild Anemia	Moderate Anemia	Severe Anemia
Non-pregnant women (≥15 years)	≥12	11–11.9	8–10.9	<8
Pregnant women	≥11	10–10.9	7–9.9	<7
Men (≥15 years)	≥13	11–12.9	8–10.9	<8

**Table 2:** Definition of iron deficiency (ID) based on serum ferritin (SF) levels, in accordance with inflammation biomarkers [19].

CRP Status	Sex	SF for ID	Sensitivity	Specificity
≤5 mg/L (no inflammation)	WHO cut-off	<12 µg/L (<5 years) <15 µg/L (>5 years)		
	Proposed cut-off	<80 µg/L	93%	96%
	Male	<77 µg/L	100%	100%
	Female	<68 µg/L	86%	91%
>5 mg/L (inflammation present)	Proposed cut-off	<200 µg/L +TSAT <13%	78%	92%
	Male	<373 µg/L	100%	68%
	Female	<146 µg/L	80%	75%

**Table 3:** Transferrin Saturation (TSAT) Reference Table.

TSAT (%)	Interpretation	Clinical Implication
<20%	Iron Deficiency	May indicate iron deficiency anemia; further evaluation needed
20–50%	Normal Range	Sufficient iron transport and storage
>50%	Possible Iron Overload	May suggest conditions like hemochromatosis or iron toxicity
45–50%	Caution Zone (Upper Normal Limit)	Could warrant monitoring in individuals with chronic disease

**Table 4:** PICO Framework for Evaluating Preoperative Anemia Management in M0-Staged CRC Surgery.

PICO Element	Description
**Population**	Adults (≥18 years) diagnosed with non-metastatic (M0-staged) colorectal cancer, scheduled for elective surgical resection
**Intervention**	Intravenous (IV) iron supplementation
**Comparator**	Oral iron supplementation
**Outcomes**	Perioperative and postoperative metrics, including: Hematologic recovery (e.g., hemoglobin level, ferritin, transferrin saturation) Need for blood transfusion Surgical access type and resectability Incidence of postoperative complications Length of hospital stay and ICU admissions Health-related quality of life, fatigue, and recovery metrics

**Table 5:** Assessment of Methodological Quality Using Jadad Scoring Criteria.

Study	Randomization Described	Appropriate Randomization Method	Double-Blinding Described	Appropriate Blinding Method	Withdrawals and Dropouts Reported	Jadad Score
Fung et al., 2022 [28]	Yes (+1)	Yes (+1)	Yes (+1)	Yes (+1)	Yes (+1)	5/5
Keeler et al., 2017 [29]	Yes (+1)	Yes (+1)	No (0)	Not applicable (0)	Yes (+1)	3/5
Talboom et al., 2023 [30]	Yes (+1)	Yes (+1)	No (0)	Not applicable (0)	Yes (+1)	3/5
Christodoulakis et al., 2005 [31]	Yes (+1)	Unclear (0)	No (0)	Not applicable (0)	Yes (+1)	2/5

**Table 6:** Risk of Bias Evaluation Using RoB2 Tool.

Study	RoB2: Randomization Bias	RoB2: Intervention Bias	RoB2: Missing Data Bias	RoB2: Outcome Measurement Bias	RoB2: Reporting Bias	RoB2: Overall Risk
Fung et al., 2022 [28]	Low Risk	Low Risk	Low Risk	Low Risk	Low Risk	Low Risk
Keeler et al., 2017 [29]	Low Risk	Some Concerns	Low Risk	Low Risk	Low Risk	Low Risk
Talboom et al., 2023 [30]	Low Risk	Some Concerns	Low Risk	Low Risk	Low Risk	Low Risk
Christodoulakis et al., 2005 [31]	Some Concerns	Low Risk	Low Risk	Some Concerns	Low Risk	Some Concerns

**Table 7:** Study Characteristics and Key Outcomes in Preoperative Anemia Management Trials.

Study	Design	Country	Population	Groups	Intervention vs. Comparator	Time of Treatment Initiation Before Surgery	Primary Outcome	Secondary Outcomes	Key Findings and Conclusions
Keeler et al., 2017 [29]	Multicentre RCT (Superiority Trial)	United Kingdom	116 Adults with CRC & anemia	IV iron: 55, Oral iron: 61	IV ferric carboxymaltose vs. Oral ferrous sulfate	Median 14 days before surgery	Mean volume of perioperative blood transfusion	Change in Hb, ferritin, transferrin saturation, anemia status at surgery, complications, length of stay, and mortality	No difference in transfusions. However, IV iron was safe and significantly better at raising Hb, ferritin, and correcting anemia before surgery.
Talboom et al., 2023 [30]	Multicentre RCT (Superiority Trial)	Netherlands and Italy	202 Adults with CRC and iron deficiency anemia	IV iron: 96, Oral iron: 106	IV ferric carboxymaltose vs. Oral ferrous fumarate	Treatment began a median of 14 days (IQR 11–22) before surgery for intravenous iron and 19 days (IQR 13–27) for Oral iron.	% of patients with normalized Hb before surgery	Hb change over time, iron store restoration (ferritin, TSAT), complications (Clavien-Dindo, CCI), transfusions, ICU admissions, QoL, fatigue, mortality	No difference in Hb normalization before surgery. However, IV iron was safe and led to significantly better Hb normalization 30 days after surgery (60% vs. 21%).
Fung et al., 2022 [28]	Pilot RCT (Double-blinded)	Hong Kong SAR	40 Adults with CRC and iron deficiency anemia	IV iron: 20, Usual care: 20	IV iron isomaltoside (20 mg/kg, max 1000 mg) vs. no iron therapy	Median 23 days (range 15–31)	Change in Hb and ferritin from baseline to pre-surgery	Transfusions, complications (Clavien-Dindo), QoR-15, DAH30, ICU admission, readmission	IV iron successfully raised Hb and ferritin. Transfusion rates were lower (though not statistically significant in this small study).
Christodoulakis et al., 2005 [31]	RCT (Open-label, 3-arm)	Greece	204 Anemic patients for colorectal surgery	Epoetin 150 IU/kg (69), Epoetin 300 IU/kg (67), Control Iron alone (68)	Epoetin alfa + iron vs. iron alone	10 days before surgery	Reduction in transfusion requirements	Hb, Hct, ferritin, TSAT, reticulocyte count, adverse events	High-dose Epoetin + Iron significantly reduced transfusions and raised Hb. The lower Epoetin dose was not effective.

**Table 8:** Surgical Approaches and Tumor Resection Sites in Colorectal Cancer Trials.

Study	Groups (*n*)	Surgery Type (Open/Lap)	Specific Resection Sites
Talboom et al., 2023 [30]	IV Iron (96), Oral Iron (106)	91% Laparoscopic overall, IV: 86 Lap/10 Open, Oral: 98 Lap/8 Open	- Right Hemicolectomy: IV 64/Oral 72 - Left Hemicolectomy: IV 14/Oral 14 - Low Anterior Resection: IV 11 /Oral 11 - Other (e.g., subtotal colectomy, sigmoid): IV 7/Oral 9
Fung et al., 2022 [28]	IV Iron (20), Usual Care (20)	IV: 14 Lap/6 Open, Control: 18 Lap/2 Open	- Rectosigmoid: IV 11/Control 12 - Non-rectosigmoid: IV 6/Control 7 - Synchronous Tumors: IV 3/Control 1
Keeler et al., 2017 [29]	IV Iron (55), Oral Iron (61)	IV: 26 Lap/22 Open/5 Converted, Oral: 30 Lap/23 Open/4 Converted	- Right Hemicolectomy: IV 29/Oral 35 - Extended RH: IV 5/Oral 5 - Left Hemicolectomy: IV 1/Oral 5 - Sigmoid/Anterior/Subtotal/Hartmann’s/APE: multiple small counts - No Resection: IV 2/Oral 4
Christodoulakis et al., 2005 [31]	EPO150 (69), EPO300 (67), Control (68)	Not specified	- Tumor Location Only: Left: ~30–36% Right: ~28–32% Rectal: ~34–37% Other: ~2%

## Data Availability

No new data were created or analyzed in this study.

## Supplementary Materials

The following supporting information can be downloaded at: https://www.mdpi.com/article/10.3390/cancers17223689/s1, Table S1: PRISMA_2020_checklist. Reference [36] is cited in Supplementary materials.
